# Transition of care in stroke patients discharged home: a single-center prospective cohort study

**DOI:** 10.1186/s12913-021-07347-7

**Published:** 2021-12-18

**Authors:** M. J. de Mooij, I. Ahayoun, J. Leferink, M. J. Kooij, F. Karapinar-Çarkit, R. M. Van den Berg-Vos

**Affiliations:** 1 Department of Neurology, OLVG, Jan Tooropstraat 164, Amsterdam, 1061 the Netherlands; 2 Department of Clinical Pharmacy, OLVG, Amsterdam, the Netherlands; 3 General Practitioner practice Rustenburg, Amsterdam, the Netherlands; 4 Community Pharmacy Koning, Amsterdam, the Netherlands; 5 Department of Neurology, Amsterdam UMC, Academic Medical Center, Amsterdam, the Netherlands

**Keywords:** Ischemic stroke, Continuity of care, Information transfer, Discharge letter, Medication overview

## Abstract

**Introduction:**

Approximately two-thirds of the patients admitted to the hospital with an ischemic stroke are discharged directly home. Discontinuity of care may result in avoidable patient harm, re-admissions and even death. We hypothesized that the transfer of information is most essential in this patient group since any future care for these patients relies solely on the information that is available to the care provider responsible at that time.

**Aim:**

The objective of this study was to evaluate the continuity of transmural care in ischemic stroke patients by assessing 1) the transfer of clinical information through discharge letters to general practitioners (GPs), 2) subsequent documentation of this information and early follow-up by GPs and 3) the documentation of medication-related information in discharge letters, at GPs and community pharmacies (CPs).

**Methods:**

This prospective cohort study was conducted from September 2019 through March 2020 in OLVG, Amsterdam, the Netherlands, in patients with a first stroke discharged directly home. Outcome measures were derived from national guidelines and regional agreements. Results were analyzed using descriptive analysis.

**Results:**

A total of 33 patients were included. Discharge letters (*n* = 33) and outpatient clinic letters (*n* = 24) to GPs contained most of the essential items, but 16% (*n* = 9) of the letters were sent in time. GPs (*n* = 31) infrequently adhered to guidelines since 10% (*n* = 3) of the diagnoses were registered using the correct code and 55% (*n* = 17) of the patients received follow-up shortly after discharge. Medication overviews were inaccurately communicated to GPs since 62% (*n* = 150) of all prescriptions (*n* = 243) were correctly noted in the discharge letter. Further loss of information was seen as only 39% (*n* = 95) of all prescriptions were documented correctly in GP overviews. We found that 59% (*n* = 144) of the prescriptions were documented correctly in CP overviews.

**Conclusion:**

In this study, we found that discontinuity of care occurred to a varying extent throughout transmural care in patients with a first stroke who were discharged home.

## Background

Approximately two-thirds of the patients admitted to the hospital with an ischemic stroke are discharged directly home [1]. These patients no longer receive continuous care from inpatient healthcare professionals as they are transferred back to primary care. It is known that continuity of care contributes to a better quality of care [2]. Continuity of care is defined as the extent to which a sequence of healthcare services is perceived as coherent, connected and consistent with the patient’s health needs and personal circumstances [2]. To guarantee continuity of care in this group of patients, timely and accurate communication to the next care providers is essential.

Numerous studies report about factors causing discontinuity in the transfer of information in patients discharged from hospital to home. First, clinical information to general practitioners (GPs) and community pharmacists (CPs) is often sent late and can be incomplete [3–6]. Discontinuity was also shown in the transfer of medication-related information to GPs and CPs, which could result in medication-related problems [3, 5–9].

Second, studies have shown that information is also lost at the level of the GP and CP. Regarding medication related information, studies show that not all information stated in discharge letters or discharge prescriptions is incorporated in the information systems of GPs and CPs [5, 7, 8]. These disruptions in the care continuum may result in avoidable readmissions and even death [10–12].

In recent years, two national guidelines were developed to improve communication in care transitions in the Netherlands. One guideline aims to improve communication between medical specialists and GPs [13]. The other aims to improve the transfer of medication-related information between hospitals and other care providers [14]. Also, to further improve and guarantee the continuity of care in several patient groups including stroke, a Transmural Platform (TP) was founded on a regional level in Amsterdam. The TP is a multidisciplinary board, which develops, implements and secures transmural working agreements [15]. Besides the usual communication of cardiovascular risk factors and treatment targets to GPs, the transmural working agreements for subacute stroke care add that the GP should visit the patient at home directly after discharge from the hospital, or should make an appointment for the patient to visit the GP practice. After care at the outpatient clinic is finished, the GP is responsible for the management of cardiovascular risk factors.

It is currently unknown to what extent these previously mentioned guidelines and working agreements are implemented and followed through in current stroke practice. Previous studies on continuity of care in stroke patients have often focused solely on the transfer of information from hospitals to GPs or from hospitals to CPs. In contrast to previous studies, we aim to evaluate the continuity of transmural care in stroke patients who are discharged home more comprehensively.

Patients who are discharged home no longer receive continuous care from an inpatient healthcare professional. Therefore, we hypothesized that the transfer of information is most essential in this patient group, since any future care for these patients relies solely on the information that is available to the care provider responsible at that time. The objective of this study was to evaluate the continuity of transmural care in first ischemic stroke patients by assessing 1) the transfer of clinical information through discharge letters to GPs, 2) subsequent documentation of this information and early follow-up by GPs and 3) the documentation of medication-related information in discharge letters, at GPs and at CPs.

## Methods

### Setting and design

We prospectively recruited participants from September 2019 to March 2020 in OLVG, a non-academic teaching hospital in Amsterdam, the Netherlands. Patients, 18 years and older, were deemed eligible for inclusion when discharged home after admission to OLVG with a first ischemic stroke. We excluded patients with a diagnosis of intracerebral haemorrhage or when patients were discharged to a nursing home or inpatient rehabilitation centre. To be able to obtain written informed consent, we also excluded patients with insufficient proficiency of the Dutch language, diagnosis of dementia or mild cognitive impairment.

### Usual care

Discharge letters, including a medication overview, were computer-generated from in-hospital electronic health records (EHRs) and edited by a resident or physician assistant before approval by a neurologist. These letters were then sent digitally to GPs by a secured e-mail. Medication reconciliation took place before a patient was discharged from the hospital [16]. In our hospital, this is done by pharmacy teams who are specially trained and educated in pharmacotherapy and communication with patients about medication. The medication overview after medication reconciliation was considered the gold standard in acquiring the best possible medication history [17]. After medication reconciliation, the medication overview could be integrated into the discharge letter and discharge prescriptions were sent to community pharmacies or the hospital’s outpatient pharmacy. If the hospital’s outpatient pharmacy dispended the prescriptions, the prescriptions were registered into a nationwide medication record system [17]. If a patient did not permit to share data through the nationwide system, a fax was sent to the patient’s CP the same day. Discharge prescriptions were always sent before or around discharge in order for a pharmacy to dispense the prescriptions. Furthermore, the transmural working agreements required GPs to visit patients at home or plan a consultation at the GP practice shortly after discharge from the hospital. Usual care included follow-up at the outpatient clinic 6 weeks after discharge from the hospital. Patients were then transferred back to primary care for further follow-up.

### Data collection and classification

Patient demographics (e.g. sex, age, days admitted) were extracted from the hospital information system. National Institutes of Health Stroke Scale (NIHSS) and the Trial of Org 10,172 in Acute Stroke Treatment (TOAST) classification were noted on participation.

An overview of the evaluated items is presented in Table 1. Discharge letters and outpatient clinic letters to GPs were obtained from in-hospital EHRs. The discharge letters were obtained after patients were discharged from hospital to home. Outpatient clinic letters were obtained 6 weeks after discharge when follow-up at the outpatient clinic was finished and patients were referred back to primary care. Both letters were evaluated on timeliness and completeness. Timeliness was defined as a discharge letter that was sent within 24 h after hospital discharge and an outpatient clinic letter that was sent within 5 days after the outpatient clinic visit [13]. Completeness was defined as whether information that was present in the in-hospital EHRs was also present in the discharge letters and outpatient clinic letters (Table 1).

**Table 1:** Overview of evaluated items based on national guidelines and transmural platform recommendations

		refs
Hospital: Discharge letter and outpatient clinic letter	Logistics	[13, 15]
	•-Timely transfer to next care provider (within 24 h after discharge for the discharge letter and within 5 days for the outpatient clinic letter)	
	•-Additional briefing of GP by phone, besides discharge letter	
	Contents, presence of:	[13–15]
	•-Diagnosis	
	•-NIHSS on admission	
	•-LDL-cholesterol level	
	•-LDL-cholesterol treatment target	
	•-Blood pressure on discharge	
	•-Blood pressure treatment target	
	•-Cardiovascular and lifestyle risk factors (e.g. hypertension, smoking)	
	•-Discharge destination	
	•-Discharge letter only: prescriptions evaluated by medication reconciliation (gold standard)	
	°Name of drug (generic or brand), dosage strength per unit, dosage frequency, dosage regimen, route of administration, time of use, including stop or end date	
	°information whether medication was changed, classified as:	
	•-unchanged (a prescription that a patient already used and continued to)	
	•-started (a prescription that was newly prescribed)	
	•-changed (change in dose, frequency or schedule)	
	•-switched (exchange for another drug in the same pharmaceutical group, e.g. when simvastatin is switched for atorvastatin)	
	•-stopped (a prescription that patients used was discontinued)	
	•-Outpatient clinic letter only: cognitive and/or emotional complaints	
GP: Documentation of clinical information and patient follow-up by GPs	Logistics	[13]
	•-Discharge letter received as stated by GP	
	Documentation	[18]
	•-Correct registration of diagnosis (ICPC-code K90.03 (ischemic stroke))	
	Patient follow-up	[15]
	•-Home visits or phone calls performed by GP	
	•-Practice visits by patients	
	Cardiovascular risk management	[15]
	•-Blood pressure was measured	
	•-Patient inclusion in CVRM program	
Medication: Documentation of medication-related information in discharge letters, at GPs and at CPs	List of prescriptions, contents compared to gold standard	[14]
	•-Name of drug (generic or brand), dosage strength per unit, dosage frequency, dosage regimen, route of administration, time of use, including stop or end date	
	•-Documentation which medication was discontinued	

*CP* Community pharmacist, *CVRM* Cardiovascular Risk Management, *ICPC-code* International Classification of Primary Care code, *LDL* Low-density lipoprotein, *GP* General practitioner, *NIHSS* National Institutes of Health Stroke Scale, *TOAST* Trial of Org 10,172 in Acute Stroke Treatment

One week after the discharge letter was sent, GPs were contacted by phone to evaluate the documentation of clinical information and patient follow-up. Following the stroke guideline of the Dutch College of General Practitioners the International Classification of Primary Care (ICPC) code K90.03 (ischemic stroke) was considered the correct code in registering the diagnosis [18].

After clinical discharge, the medication overviews after medication reconciliation (gold standard) were collected from in-hospital EHRs and all prescriptions were classified as unchanged, started, changed, switched or stopped during admission (see Table 1). Medication overviews were requested from CPs 1 week after clinical discharge and medication overviews from the GP were requested 1 week after the discharge letter was sent. In this way, CPs and GPs both had approximately 1 week to document any changes in medication in their information system.

Medication overviews from the discharge letter, GP and CP were compared with the list of prescriptions from the gold standard. The documentation of prescriptions in the discharge letter, GP overviews and CP overviews was scored as “complete” when the prescriptions corresponded to the gold standard on all evaluated items (see Table 1). If any element was missing (e.g. a frequency), the documentation of information was scored as “incomplete”. If the prescription was not noted at all, it was scored as “no documentation”. For *changed and switched* prescriptions, it should be clear that the first prescription was discontinued (e.g. when perindopril dose was increased from 2 to 4 mg, perindopril 2 mg needed to be discontinued). Documentation was necessary regarding which medication was discontinued. Changes in prescriptions from generic to branded or branded to generic medication were allowed and were not scored as a discrepancy.

Two investigators (MM and IA) independently assessed the discharge letter, outpatient clinic letter and medication overviews and any differences in the assessment were noted. Cases were discussed with a supervisor (FK) if consensus could not be reached. A sub-analysis was performed for the transfer of information of clinically relevant medication for the secondary prevention of stroke (i.e. platelet aggregation inhibitors, oral anticoagulants, antihypertensive drugs, statins and ezetimibe, and proton pump inhibitors).

### Statistical methods

Data was analysed with IBM SPSS Statistics for Windows, version 22.0 (IBM, Chicago) using descriptive analysis. To describe continuous and discrete variables with a normal distribution, mean and standard deviation were used. For continuous and discrete variables without a normal distribution, median and an interquartile range were used. Frequencies and percentages were used to describe dichotomous, nominal and ordinal data.

## Results

### Participants

**Fig. 1:**
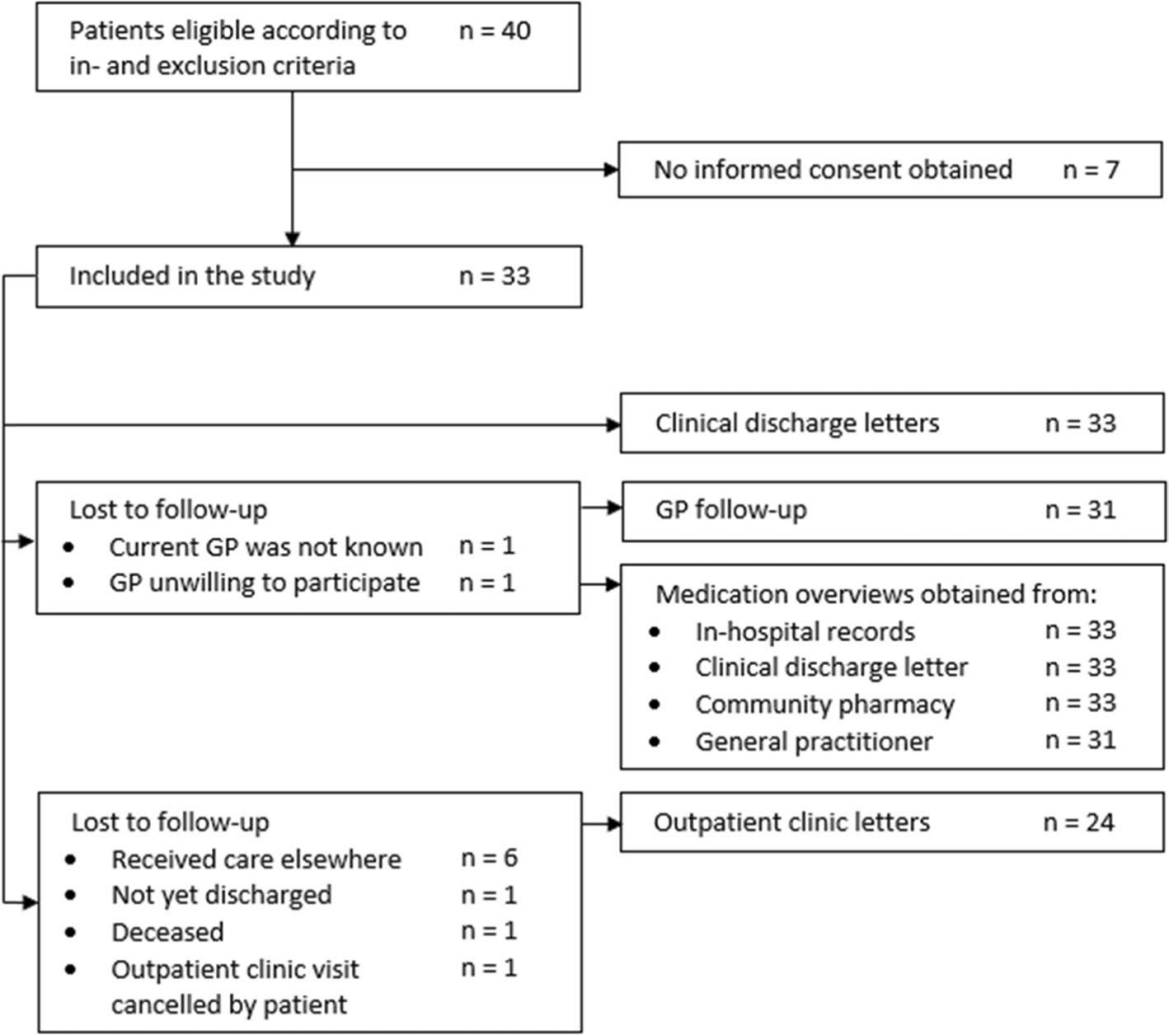
Study flow diagram

Forty patients were deemed eligible for inclusion. Seven patients did not provide informed consent. A total of 33 patients were included in the study. Details about loss to follow-up are shown in Fig. 1. The mean age of the participants was 66 years (± 13.8 SD) of which 52% were male. The median NIHSS on admission was 2 (IQR 1). Further details about patient characteristics are listed in Table 2.

**Table 2:** Patient characteristics

Patients, n (%)	33	
Male	17	52%
Female	16	48%
Age, years (mean, ±SD)	66.1	±13.8
Days admitted (median, IQR)	2	1
Readmissions, n (%)	3	9%
Death, n (%)	1	3%
NIHSS on admission (median, IQR)	2	1
TOAST classification, n (%)		
I. Large vessel atherosclerosis	5	15%
II. Cardio embolism	5	15%
III. Small vessel occlusion (lacune)	9	27%
IV. Stroke of other determined aetiology	1	3%
V. Stroke of undetermined aetiology	13	39%

*IQR* Interquartile range, *NIHSS* National Institutes of Health Stroke Scale, *SD* Standard deviation, *TOAST* Trial of Org 10,172 in Acute Stroke Treatment

### Discharge letters

A total of 33 discharge letters and 24 outpatient clinic letters were assessed (Table 3). Six (18%) of the discharge letters and three (13%) of the outpatient clinic letters were sent according to the guideline within 1 and 5 days, respectively. The discharge letters were sent to the GP on a median of 4 (IQR 4) days post-discharge. The outpatient clinic letter was sent on a median of 9 (IQR 6) days after the outpatient clinic visit. All discharge and outpatient clinic letters contained the diagnosis. Low-density lipoprotein (LDL) levels, LDL-targets and blood pressure levels were found to be reported in every outpatient clinic letter. Target values for blood pressure were communicated less frequently (42%). All discharge letters stated the discharge destination and all outpatient clinic letters stated that care was transferred back to the GP.

**Table 3:** The transfer of information through discharge letters and outpatient clinic letters

	Discharge letter		Outpatient clinic letter	
Logistics				
Letters sent to GP	33		24	
Sent in time according to guideline	6	18%	3	13%
Time-to-send, days (median, IQR)	4	IQR 4	9	IQR 6
Hospital additionally briefed GP via telephone call	3	10%	0	
Contents, presence of:				
Diagnosis	33	100%	24	100%
NIHSS on admission	29	94%	0	
LDL-cholesterol level	33	100%	24	100%
LDL-cholesterol target	5	15%	24	100%
Blood pressure on discharge	15	45%	24	100%
Blood pressure target	0	0%	10	42%
Discharge destination	33	100%	24	100%
Cognitive/emotional complaints			16	67%
Does have complaints and asks GP to follow-up			11	
Does not have complaints			5	

*GP* General practitioner, *IQR* Interquartile range, *LDL* Low-density lipoprotein, *NIHSS* National Institutes of Health Stroke Scale

On discharge, the in-hospital EHRs mentioned a total of 75 cardiovascular and lifestyle risk factors of which the discharge letter stated 57 (76%). In outpatient clinic letters, after post-stroke care in the hospital was finished, this number increased to 86 cardiovascular risk factors. Recommendations to the GP for the treatment of these cardiovascular risk factors were stated in 2 (4%) and 29 (34%) of discharge letters and outpatient clinic letters, respectively.

### Documentation and follow-up by GPs

Data on the documentation of information from the discharge letter and follow-up of patients by GPs were retrieved from 31 GPs (Table 4). Two GPs (6%) stated that the discharge letter was not received. A diagnosis was registered by GPs in 26 (84%) of the patients and was done according to GP guidelines in 3 (10%) patients. Over half of all the patients (55%) received follow-up by GPs in the first few weeks following discharge from the hospital.

**Table 4:** Documentation and follow-up actions by General practitioners

	n	(%)
GPs participating	31	
Discharge letter received	29	94%
Diagnosis registered	26	84%
Stroke	20	65%
Ischemic stroke, according to guideline	3	10%
TIA	1	3%
Other	2	6%
Patients received any follow-up by GP	17	55%
GP called patient	10	32%
GP visited patient at home	5	16%
Patient visited GP practice	4	13%
GP included patient in CVRM program	10	32%
GP or assistant measured blood pressure	3	10%

*CVRM* Cardiovascular Risk Management, *GP* General Practitioner, *TIA* Transient Ischemic Attack

### Transfer of medication-related information

A total of 243 prescriptions were subtracted for 33 patients from the in-hospital EHRs after medication reconciliation at discharge was performed. Discharge letters documented 150 (62%) of the prescriptions completely, whereas 60 (25%) of the prescriptions were incompletely documented and 33 (14%) were not documented.

The CPs correctly documented 144 of the 243 (59%) prescriptions in their systems, with 56 (23%) of the prescriptions incorrectly documented and 43 (18%) not documented.

The GPs correctly documented 95 of the 243 (39%) prescriptions in their systems, with 58 (24%) of the prescriptions incorrectly documented and 63 (26%) not documented. Due to 2 missing GP medication overviews, the documentation of 27 (11%) prescriptions by the GP was not known. In the sub-analysis of the clinically relevant medication for the secondary prevention of stroke, similar percentages of documentation of these prescriptions were found. The in-detail data of these analyses can be found in Table 5.

**Table 5:** Completeness of medication-related information in discharge letter and community pharmacy and general practitioner medication overviews

	Discharge letter overviews (***n*** = 33)						Community pharmacy overviews (***n*** = 33)						General practitioner overviews (***n*** = 31)							
	Complete documentation *n*=, (%)^a^		Incomplete documentation *n*=, (%)^a^		No documentation *n*=, (%)^a^		Complete documentation *n*=, (%)^a^		Incomplete documentation *n*=, (%)^a^		No documentation *n*=, (%)^a^		Complete documentation *n*=, (%)^a^		Incomplete documentation *n*=, (%)^a^		No documentation *n*=, (%)^a^		Unknown *n*=, (%)^a^	
**Total prescriptions,** ***n*** **= 243**	**150**	**(62%)**	**60**	**(25%)**	**33**	**(14%)**	**144**	**(59%)**	**56**	**(23%)**	**43**	**(18%)**	**95**	**(39%)**	**58**	**(24%)**	**63**	**(26%)**	**27**	**(11%)**
Unchanged, *n* = 126	82	(65%)	23	(18%)	21	(17%)	92	(73%)	21	(17%)	13	(10%)	63	(50%)	24	(19%)	23	(18%)	16	(13%)
Started, *n* = 67	52	(78%)	13	(19%)	2	(3%)	43	(64%)	4	(6%)	20	(30%)	28	(42%)	7	(10%)	29	(43%)	3	(4%)
Change, *n* = 6	1	(17%)	4	(67%)	1	(17%)	2	(33%)	2	(33%)	2	(33%)	0		1	(17%)	2	(33%)	3	(50%)
Switched, *n* = 20	8	(40%)	12	(60%)	0		7	(35%)	12	(60%)	1	(5%)	2	(10%)	11	(55%)	4	(20%)	3	(15%)
Stopped, *n* = 24	7	(29%)	8	(33%)	9	(38%)	0		17	(71%)	7	(29%)	2	(8%)	15	(63%)	5	(21%)	2	(8%)
**Clinically relevant prescriptions,** ***n*** **= 121**	**86**	**(71%)**	**29**	**(24%)**	**6**	**(5%)**	**72**	**(60%)**	**23**	**(19%)**	**26**	**(21%)**	**53**	**(44%)**	**25**	**(21%)**	**34**	**(28%)**	**9**	**(7%)**
Platelet aggregation inhibitors, *n* = 42	33	(79%)	8	(19%)	1	(2%)	27	(64%)	5	(12%)	10	(24%)	16	(38%)	6	(14%)	17	(40%)	3	(7%)
Oral anticoagulants, *n* = 5	4	(80%)	0		1	(20%)	3	(60%)	0		2	(40%)	3	(60%)	2	(40%)	0		0	
Antihypertensive drugs, *n* = 35	26	(74%)	6	(17%)	3	(9%)	27	(77%)	4	(11%)	4	(11%)	26	(74%)	3	(9%)	4	(11%)	2	(6%)
Statins, *n* = 32	21	(66%)	10	(31%)	1	(3%)	10	(31%)	12	(38%)	10	(31%)	6	(19%)	13	(41%)	10	(31%)	3	(9%)
Ezetimibe, *n* = 1	1	(100%)	0		0		1	(100%)	0		0		1	(100%)	0		0		0	
Proton pump inhibitors^b^, *n* = 6	1	(17%)	5	(83%)	0		4	(67%)	2	(33%)	0		1	(17%)	1	(17%)	3	(50%)	1	(17%)

^a^Percentages are calculated relative to in-hospital overviews after medication reconciliation, which is considered the gold standard

^b^Only esomeprazole and omeprazole were assessed due to their interaction with clopidogrel

## Discussion

In this study, we evaluated the continuity of transmural care in stroke patients discharged to home and found that discontinuity occurred to a varying extent in items that are essential according to national guidelines and regional agreements. Discontinuity in one setting was found to impact the continuity further in the care continuum. The discharge letter and outpatient clinic letter to GPs contained most of the evaluated items, but only 16% of the letters were sent in time. Moreover, GPs registered only 10% of the diagnoses correctly and 45% of the patients received follow-up shortly after discharge. In general, medication overviews were inaccurately communicated to GPs since 62% of all prescriptions were correctly noted in the discharge letter. Further loss of information was seen as only 39% of all prescriptions were documented correctly in GP overviews. We found that 59% of the prescriptions were documented correctly in CP overviews. Similar percentages were seen when we assessed clinically relevant medication for the secondary prevention of stroke separately. To improve continuity of care, in depth knowledge is required in understanding how care is organized, even when EHRs are in place, and which human factors play a role.

First, discharge letters are computer-generated from in-hospital EHRs and subsequently edited by a resident and approved by a neurologist before they are sent to the GP. This process of editing and approval may result in letters being delayed. The patients included in this study were mostly short stay subjects, which is a possible explanation for the poor amount of discharge letters being sent within 24 h after discharge. Moreover, some of the evaluated items were not always communicated to the GP. For example, the presence of emotional or cognitive complaints was not provided in all outpatient clinic letters and might have been left out because these complaints were not present. However, it is still useful for the GP to know that specific symptoms are absent on patient follow-up and these should therefore be included in the letters to the GP.

The incompleteness of prescriptions in discharge letters resulted from residents not including the medication overview as generated after medication reconciliation (gold standard). When this altered overview was included in the discharge letter, information on dose changes or stopped prescriptions was sometimes omitted. This comprised a large part of the incorrectly documented and not documented prescriptions in the discharge letter. Since GPs were provided with incomplete information from medication overviews in the discharge letter, the amount of correctly documented prescriptions in GP overviews was even lower. Moreover, upon requesting the medication overviews from the CP, the nationwide medication record system is not accessed automatically and requires a manual action from the CP. As a result, the list of prescriptions was not always up-to-date as it was based on previously documented data in the CP’s EHR. Patients may receive suboptimal treatment when they are readmitted in another hospital and GPs or CPs provide incomplete or incorrect information (e.g. when a stroke patient is recently started on oral anticoagulation for paroxysmal atrial fibrillation and presents with an aspiration pneumonia in another hospital unaware of this recent prescription). The compatibility between the hospital’s, GP’s and CP’s healthcare software is also limited. This may lead to errors since the CP and GP often have to document the changed prescriptions manually. Overall, our study shows that a part of the information was lost every time information was transferred.

The percentage of clinical discharge letters sent within 24 h after discharge was slightly less in our study (18%) than reported in other studies (24–26%) [7, 19]. A review by Kripalani et al. reported that discharge letters were missing the main diagnosis (18%), physical findings (11%), diagnostic test results (38%), discharge medications (21%) and follow-up plans (14%) [4]. Since there is no gold standard in assessing timeliness and completeness of discharge letters, and outcomes vary widely, it is difficult to compare our data to other studies. Overall, our study confirms data from other healthcare systems that discharge and outpatient clinic letters are incomplete to a varying extent for the items evaluated.

In this study, almost half of the patients (45%) did not receive follow-up by GPs in the first few weeks after discharge. Misky et al. reported a comparable percentage (51%) and found that discharged internal medicine patients lacking GP follow-up were 10 times more likely to be readmitted within 30 days of discharge [20]. However, early follow-up by GPs was not found to reduce readmission or mortality rates in another study [21].

The percentage of correctly documented prescriptions in discharge letters was similar in our study (62%) to percentages found in earlier studies (47–85%) [5, 7]. However, the correct documentation of changed prescriptions (start, dose change, switch and stop combined) by CPs was lower in our study (41%) when compared to a similar Dutch study (56%) [8]. Moreover, our study also reports lower percentages of correct documentation of changed prescriptions and unchanged prescriptions by GPs (39 and 50%, respectively) than a previous Dutch study (50 and 75%, respectively) [5]. These results confirm that the documentation of medication-related information is often incomplete, despite the introduction of guidelines and recommendations for the transfer of medication-related information.

### Strengths and limitations

A key strength of this study was that, to our knowledge, this is the first study that incorporates continuity of care on clinical information and medication use and shows the discontinuity in discharge letters, in GP practice and CP practice together. Another strength was that we assessed the items quantitatively and qualitatively (e.g. presence and accuracy of communicating discharge prescriptions).

Some limitations have to be discussed. First, the sample size in our study was small. However, the results we found were similar as found in previous larger studies before the new guideline was implemented [5, 7, 8]. Therefore, we do not believe that a larger sample size would change our conclusions about discontinuity in the care continuum. Second, our data may show an overestimation of errors in the transfer of medication-related information because we used strict criteria to score completeness. However, using these strict criteria left little room for interpretation and improved the reproducibility of the study. Third, this study focused on patients discharged home limiting the generalizability to other patient groups. When taking the exclusion of patients with limited proficiency of the Dutch language, cognitive impairment or dementia into account, it is worrisome that these patient groups will likely encounter even more difficulties in the transition from hospital to home. Fourth, he subject of continuity of care inherently depends strongly on the national or regional organization of healthcare and thus generalizability to other healthcare systems may be limited.

### Possible solutions to improve continuity of care

The data from this study provide insight into the continuity of stroke care in an exploring manner and identify areas that could be the aim for improvement strategies to achieve better post-stroke care. We think multiple interventions can significantly improve continuity of care in stroke patients discharged home.

Troude et al. showed improvement of both completeness and timeliness of discharge documentation after educating physicians and after the introduction of a fixed-format discharge letter with automatically generated fields by the EHR system. However, discharge medications and treatments were still only present in 44% of letters after completion of the intervention and these were not assessed qualitatively [22].

A recent study by Pedersen et al. acknowledged the gap between secondary and primary care in which omissions in follow-up topics in the discharge letters were found to contribute the most [23]. Adding the recommendations for follow-up from the regional agreements in the discharge letter thus may increase compliance by GPs.

Increasing the patient’s education and participation by improving accessibility to medical information and their discharge information through online patient portals can be a partial solution to further improve continuity and quality of care [24].

Currently, several solutions for these poor results are being implemented in the Netherlands. National information standards are being developed that make it possible to communicate with any EHR of any healthcare provider. In the Netherlands, all healthcare providers use EHRs. However, these are not compatible with each other. Adopting changes to these EHR systems to improve the exchangeability of information is often limited by technical limitations, high cost and privacy legislation and is therefore still in development. However, Boockvar et al. found, in a system with an EHR and without, that the amount of patients with incomplete medication lists did not differ [25]. We think that the lack of compatibility of EHR systems is important but the human factor in how EHR systems are used also plays a role. Continuity of care is dependent on how healthcare providers use EHRs and whether healthcare providers update information continuously and act on guidelines (e.g. performing a home visit or using e-health solutions). Studies show that for continuity of care and reducing patient harm collaboration is needed between settings and that bridging interventions have more impact compared to solitary interventions in one setting [26–28]. Therefore, also complex interventions need to be embedded focusing on discharge management in hospitals and more follow-up by primary care providers for vulnerable patients. A large multicenter study assessing rates of adverse events caused by discontinuity of care before and after the implementation of a structured transitional care program may provide the answer to this question. Furthermore, we would like to encourage other research groups in other countries to evaluate the transition of care in their national healthcare systems.

## Conclusion

In conclusion, this study showed that discontinuity of care occurs to a varying extent throughout transmural care in ischemic stroke patients discharged home, and that discontinuity in one level impacts the discontinuity further in the care continuum.

## Data Availability

The datasets used and/or analyzed during the current study are available from the corresponding author on reasonable request.

## Abbreviations

CP: Community pharmacy
CVRM: Cardiovascular Risk Management
EHR(s): Electronic Health record(s)
GP: General practitioner
ICPC: International Classification of Primary Care
IQR: Interquartile range
LDL: Low-density lipoprotein
NIHSS: National Institutes of Health Stroke Scale
SD: Standard deviation
TIA: Transient Ischemic Attack
TOAST: Trial of Org 10,172 in Acute Stroke Treatment
TP: Transmural platform
